# Supplementary material for: The Solid-State Synthesis of BiOIO3 Nanoplates with Boosted Photocatalytic Degradation Ability for Organic Contaminants
Source: Molecules. 2023 Apr 24;28(9):3681. doi: 10.3390/molecules28093681 (PMC10180272; doi:10.3390/molecules28093681)
Supplement: Supplementary file 1 [file molecules-28-03681-s001.zip › molecules-2326115-supplementary.pdf]

Supporting Information for

The Solid-State Synthesis of $BiOIO_3$ Nanoplates with Boosted Photocatalytic Degradation Ability for Organic Contaminants

Jia Li [†], Jing Xie [*,†], Xiaojing Zhang, Enhui Lu and Yali Cao [*]

State Key Laboratory of Chemistry and Utilization of Carbon Based Energy Resources, College of Chemistry, Xinjiang University, Urumqi 830046, China

* Correspondence: xiejing@xju.edu.cn (J.X.); caoyali523@163.com (Y.C.); Tel.: +86-991-8583083 (Y.C.); Fax: +86-991-8588883 (Y.C.)

† These authors contributed equally to this work.

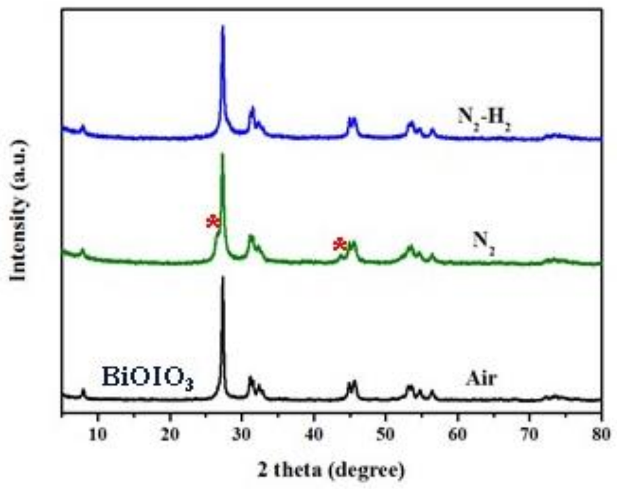

Figure S1 XRD patterns of $BiOIO_3$ synthesis with different calcination atmosphere.

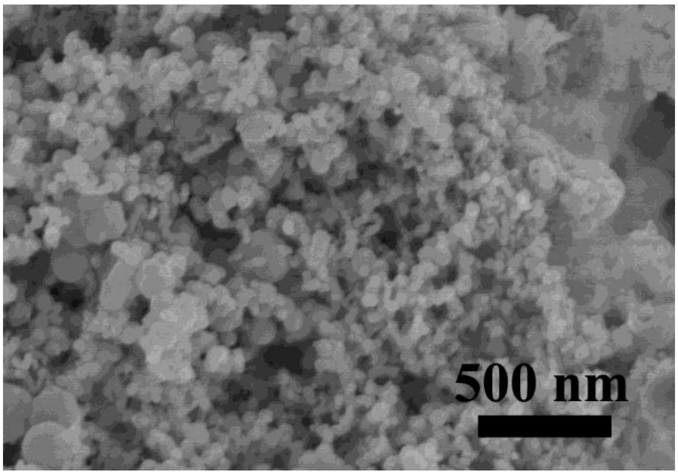

Figure S2 FESEM image of the precursor.

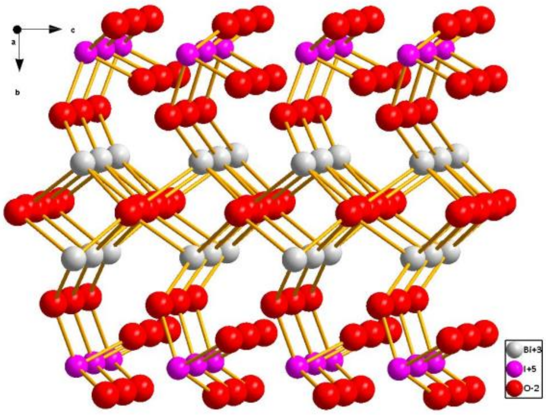

Figure S3 The crystal structure of $BiOIO_3$.

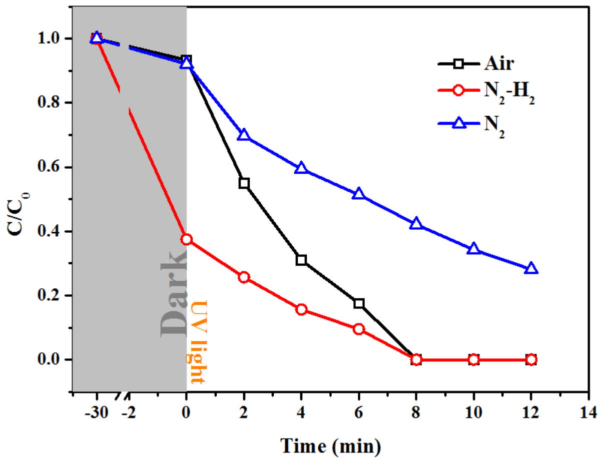

Figure S4 Degradation efficiencies of MO over $BiOIO_3$ synthesis with different calcination atmosphere under UV light irriadation.

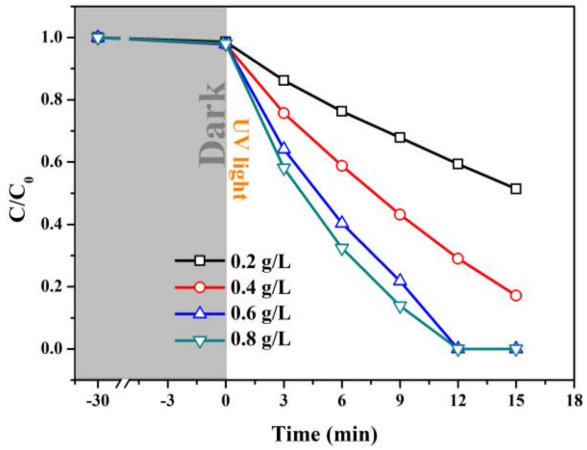

Figure S5 Degradation efficiencies of MO by different concentrations of $BiOIO_3$.

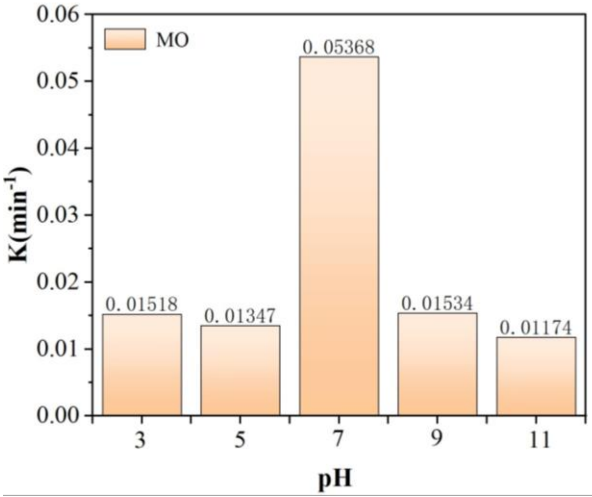

Figure S6 Degradation efficiencies of MO over $BiOIO_3$ in different pH.

Table S1 The comparison of the degradation for MO in this study with other investigations.

Catalyst	The concentration of MO	Catalyst dosage (mg)	Irradiation time (min)	Removal rate (%)	Ref.
$BiOIO_3$	10 mg/L	25	10	100	This work
$BiOIO_3$	200 mg/L	100	12	92%	[S1]
10% Co-ZnO	100 mg/L	50	120	93	[S2]
SbSI nanowires	30 mg/L	25	10	97	[S3]
TiO_2 nanotubes network	20 mg/L	-	120	80	[S4]
$Ca_{0.8}$-β-In_2S_3	10 mg/L	30	30	98	[S5]
CuPd/ZnO	40 mg/L	50	45	95	[S6]
porous graphene/ZnO	13 mg/L	-	150	100	[S7]

[S1] Wang, W.J.; Huang, B.B.; Ma, X.C.; Wang, Z.Y.; Qin, X.Y.; Zhang, X.Y.; Dai, Y.; Whangbo, M.H. Efficient separation of photo-generated electron-hole pairs by the combination of a heterolayered structure and internal polar field in pyroelectric BiOIO3 nanoplates. *Chem. Eur. J.* **2013**, 19, 14777-14780.

[S2]Adeel, M.; Saeed, M.; Khan, I.; Muneer, M.; Akram, N. Synthesis and characterization of Co-ZnO and evaluation of its photocatalytic activity for photodegradation of methyl orange. *ACS Omega*. **2021**, 6, 1426-1435.

[S3] Wang, R.H.; Wang, Y.N.; Zhang, N.N.; Lin, S.; He, Y.J.; Yan, Y.J.; Hu, K.; Sun, H.J.; Liu, X.F. Synergetic piezo-photocatalytic effect in SbSI for highly efficient degradation of methyl orange. *Ceram. Int*. **2022**, 48, 31818-31826.

[S4] Yang, J.; Du, J.; Li, X.; Liu, Y.; Jiang, C.; Qi, W.; Zhang, K.; Gong, C.; Li, R.; Luo, M.; Peng, H. Highly hydrophilic TiO_2 nanotubes network by alkaline hydrothermal method for photocatalysis degradation of methyl orange. *Nanomaterials-Basel*. **2019**, 9, 526-539.

[S5] Yao, W.; Chen, Y.F.; Li, J.L.; Yang, J.; Ren, S.; Liu, W.C.; Liu, Q.C. Photocatalytic degradation of methyl orange by Ca doped β-In_2S_3 with varying Ca concentration. *Res. Chem. Intermediatr.* **2022**, 48, 1813-1829.

[S6] Sun, H.; Lee, S.Y.; Park, S.J. Bimetallic CuPd alloy nanoparticles decorated ZnO nanosheets with enhanced photocatalytic degradation of methyl orange dye. *J. Colloid Interface Sci.* **2022**, 629, 87-96.

[S7] Wang, L.; Li, Z.; Chen, J.; Huang, Y.; Zhang, H.; Qiu, H. Enhanced photocatalytic degradation of methyl orange by porous graphene/ZnO nanocomposite. *Environ. Pollut.* **2019**, 249, 801-811.
